# Editorial: Modern management of ruptured abdominal aortic aneurysm

**DOI:** 10.3389/fcvm.2024.1487836

**Published:** 2024-09-12

**Authors:** Harm Pieter Ebben, Petar Zlatanovic, Desiree van den Hondel, Vishal Amlani, Mario Giovanni Gerardo D’Oria, Nicola Troisi, Albert Bush

**Affiliations:** ^1^ Department of Surgery, Amsterdam UMC Location Vrije Universiteit Amsterdam, Amsterdam, Netherlands; ^2^ Clinic for Vascular and Endovascular Surgery, University Clinical Centre of Serbia, Belgrade, Serbia; ^3^ Department of Surgery, Leeuwarden Medical Center, Leeuwarden, Netherlands; ^4^ Department of Vascular Surgery, Sahlgrenska University Hospital, Gothenburg, Sweden; ^5^ Department of Vascular Surgery, University Hospital of Trieste, Trieste, Italy; ^6^ Vascular and Endovascular Surgery Unit, University Hospital of Pisa, Pisa, Italy; ^7^ Division of Vascular and Endovascular Surgery, Department for Visceral-, Thoracic and Vascular Surgery, Medical Faculty Carl Gustav Carus and University Hospital, Technische Universität Dresden, Dresden, Germany

**Keywords:** RAAA, ruptured abdominal aorta aneurysm, EVAR, OSR, acute aortic syndrome, ruptured aneurysm, acute aortic surgery, aortic surgery

**Editorial on the Research Topic**
Modern Management of ruptured abdominal aortic aneurysm

Ruptured abdominal aortic aneurysms (rAAA) present one of the direst emergencies in vascular surgery, demanding immediate and decisive surgical intervention. Historically, the management of rAAA has always been accompanied with challenges, primarily due to the rapid deterioration of patients and the complex decision-making required in emergency settings. Innovations over the years have transitioned treatment from primarily open surgical repairs to more minimally invasive endovascular techniques, which have significantly altered the outcomes. Despite advancements, the treatment of rAAA continues to be complicated by factors such as the variability in patient anatomy, the timing of intervention, and the availability of appropriate surgical expertise (1). This editorial introduces four original research articles that explore the evolving paradigms in treatment, assess the impact of clinical guidelines, assess the impact of stent selection on perioperative outcomes and delve into the use of off-the-shelf devices for complex cases. It sheds light on how these developments are tackling the enduring challenges in the management of rAAA.

The transformation in the management of rAAA is characterized by significant advancements in surgical techniques, particularly the shift from open surgical repair (OSR) to endovascular aneurysm repair (EVAR). The first article offers an in-depth examination of this transition, emphasizing the procedural innovations and technological advancements that have made EVAR the preferred option (Scali and Stone). The article reviews comparative studies that demonstrate EVAR's effectiveness in reducing short-term mortality compared to OSR, alongside facilitating quicker patient recovery and shorter hospital stays. Additionally, the discussion on cost-effectiveness explores the economic implications of adopting EVAR more broadly within healthcare systems. While the initial costs associated with EVAR are higher, the article argues that these will be offset by the reduction in postoperative complications and the decrease in long-term healthcare expenditures, making EVAR not only a clinically superior option but also a financially viable one. The authors set the stage for further discussion on how these innovations can be integrated into standardized care protocols to enhance overall patient management and outcomes.

The second article provides a critical analysis of the clinical guidelines issued by the National Institute for Health and Care Excellence (NICE) for managing rAAAs (Eilenberg et al.). Employing a novel simulated target trial approach, the authors assess how these guidelines perform (hypothetically) in real-world clinical settings. The study unveils considerable variability in the effectiveness of the guidelines across different patient demographics, including age, underlying health conditions, and the severity of the aneurysm at the time of presentation. The findings highlight the limitations of a one-size-fits-all approach to guideline implementation, pointing out the discrepancies between ideal clinical outcomes and actual patient results. This raises important questions about the adaptability of current protocols to the nuanced needs of diverse patient groups. Consequently, the article advocates the need for more personalized treatment strategies but also suggests that enhancing guideline flexibility could significantly improve overall patient outcomes.

The third article focuses on the impact of different endovascular stent types on the perioperative outcomes of rAAAs based on a single center experience (Ma et al.). This study highlights how the choice of stent—specifically comparing the Gore Excluder (W.L. Gore & Associates, Inc., Flagstaff, Arizona) and Microport Hercules (Micro Port. Shanghai. China)—can influence patient outcomes, including perioperative mortality- and complication rates. This article underlines the necessity of personalized stent selection based on patient-specific factors and the potential for improving rAAA management by optimizing stent choice.

The fourth article examines urgent and emergent repair strategies for complex aortic aneurysms using an off-the-shelf branched device, focusing on a study conducted at two aortic centers (Nana et al.). Their analysis delves into the use of the t-Branch device for managing thoracoabdominal and para-renal aneurysms. The findings underscore the importance of device selection and procedural expertise in influencing patient outcomes, with urgent cases showing slightly better survival rates compared to emergent cases. This study emphasizes the potential for off-the-shelf devices to improve the management of life-threatening aneurysms, despite the inherent complexities of such urgent interventions.

These articles collectively underscore the evolving dynamics of rAAA management, illustrating the necessity of a multidisciplinary approach that merges cutting-edge technology with evidence-based clinical practices. They demonstrate the importance of integrating advanced diagnostic and procedural technologies, revising clinical guidelines (1) to reflect the latest research, and crafting patient care strategies that are as personalized as they are comprehensive.

The insights provided by these studies not only enrich our current understanding but also pave the way for innovative treatment paradigms. As we advance, the emphasis must remain on a flexible, adaptive approach that continuously incorporates new findings and technologies. This will be crucial in enhancing patient outcomes and effectively managing the complexities of rAAA. The call to action for ongoing innovation and adaptability is clear: aiming to ensure that every patient facing this critical condition receives the most effective and applicable treatment possible.
